# SARS-CoV-2 pandemia in Lombardy: the impact on family Paediatricians

**DOI:** 10.1186/s13052-020-00950-0

**Published:** 2020-12-21

**Authors:** Rinaldo Missaglia, Michael Belingheri, Laura Antolini, Monica de’ Angelis, Luca Brivio, Michele Augusto Riva, Simonetta Genovesi

**Affiliations:** 1 Family Pediatrician, ATS Brianza, SIMPeF Milan, Milan, Italy; 2 School of Medicine and Surgery, University of Milano-Bicocca, Monza, Italy; 3 Family Pediatrician, ATS Milan, SIMPeF Milan, Monica de’Angelis, Via Aosta 13, 20155 Milan, Italy; 4 Cardiologic Unit, Istituto Auxologico Italiano, IRCCS, Milan, Italy

**Keywords:** Family paediatricians, Health care workers, COVID-19, SARS-CoV-2 antibodies

## Abstract

As health care workers (HCWs) who care for children, who usually demonstrate milder symptoms than adults, family paediatricians have an increased risk of exposure to coronavirus. In April 2020, the Sindacato Medici Pediatri di Famiglia (SIMPeF), provided its members with rapid tests to detect antibodies against SARS-CoV-2; 1240 individuals, including 377 paediatricians, 108 staff members of pediatric clinics, and 755 cohabitant relatives of paediatricians, were tested in Lombardy, the most affected Italian region. The global prevalence of IgG antibodies in these individuals was 20.7%, which is higher than that of the general population and other HCWs. More than 70% of subjects with IgG antibodies presented symptoms, and 4.9% needed hospitalization. In addition, 64.2% of the study participants reported close contacts with a suspected case of COVID-19, while 72.9% of the family paediatricians reported occupational exposure to the disease. The initiative of the SIMPeF has been useful in assessing the impact of the COVID-19 pandemic on paediatric settings, as well as in raising paediatricians’ awareness of the spreading of coronavirus.

Novel coronavirus disease 2019 (COVID-19) outbreak has spread rapidly all over the world, having resulted in more than 42 million cases to date [1]. Health care workers (HCWs) have an increased risk of exposure to coronavirus, as do other individuals who work in health care settings, where they come into contact with patients [2, 3]. Nevertheless, the risk of infection for HCWs who work in a COVID-19 designated department is debated. If on the one hand the use of proper personal protective equipment (PPE) and an increased awareness may reduce the risk of contagious, on the other an incorrect application and removal of PPE and particular clinical situations (such as practice location) may increase the risk [4, 5]. Family paediatricians can be considered as a particular group of HCWs at even higher risk of contracting COVID-19 from their patients than other HCWs, due to the specific clinical features of coronavirus disease in children. Symptoms described in Italian and Chinese children are milder than those in the adult population. Furthermore, symptoms in children are usually not specific (as fever and/or cough), and many children with SARS-CoV-2 infection are asymptomatic, as can also happen in adults without comorbidities [6–8]. In the US, children with more severe symptoms (i.e. multisystem inflammatory syndrome in children associated with COVID-19 disease) have been described, but these are still relatively few cases [9, 10].

For these reasons, especially at the beginning of the outbreak, when no one knew the characteristics of this new disease, family paediatricians and their staff members may have therefore been exposed to coronavirus without adequate personal protective equipment.

The Sindacato Medici Pediatri di Famiglia (SIMPeF), provided its members who have accepted to be part of the study with rapid tests to detect IgG and IgM antibodies against SARS-CoV-2 to be administered to themselves, their collaborators and their cohabitant relatives. Therefore the study sample is made of three sub-samples, one sample of paediatricians, one sample of staff members of paediatric clinics, one sample of paediatricians relatives. To account for the study design, and the subsequent non homogeneity, the statistical analysis was performed separately in the three subsamples. Out of 844 SIMPeF members (of which 824 resident in Lombardy, the most affected Italian region), 377 agreed to participate in the study. The presence of specific antibodies was tested in vitro using the 2019-nCoV IgG/IgM Rapid Test Cassette (ACRO BIOTECH, Inc.), a lateral flow chromatographic immunoassay for the qualitative detection of antibodies in human whole blood, serum, or plasma specimen. The technical manual reports the performance characteristics [11]. For the IgG results, relative sensitivity was 100% (95% CI: 86.0–100), relative specificity was 98.0% (95% CI: 89.4–99.9), and accuracy was 98.6% (95% CI: 92.3–99.96), according to the manufacturer [11]. As to the IgM results, relative sensitivity was 85.0% (95% CI: 62.1–96.8), relative specificity was 96.0% (95% CI: 86.3–99.5), and accuracy was 92.9% (95% CI: 84.1–97.6), as described in the technical manual [11].

Data was collected through an online questionnaire available on the SIMPeF website; the participants completed this questionnaire voluntarily and reported any symptoms related to COVID-19, any contacts with possible or confirmed COVID-19 cases, and rapid test results (IgG and IgM). In some cases, especially when the rapid test was positive, a polymerase chain reaction (PCR) diagnostic test on nasal swabs was performed.

The study included 1240 individuals who lived in Lombardy, the most affected Italian region [12]. Specifically, 377 paediatricians, 108 staff members of paediatric clinics, and 755 cohabitant relatives of paediatricians were enrolled. To account for the study design, and the subsequent non homogeneity, the statistical analysis was conducted separately in the three subsamples. Table 1 presents the main characteristics of the study population. More women participated than men, and the average age of the participants was 48.3(17.0) years; the family paediatricians were older than the other subjects (Table 1).

**Table 1:** Main characteristics of study population (*N* = 1240)

Variables	Total		Paediatricians		Staff members		Cohabitant relatives	
	N	%	N	%	N	%	N	%
Age [average (DS)]	48.3 (17.0)		57.2 (7.2)		48.4 (10.1)		43.8 (19.3)	
Gender								
Male	495	39.9%	88	23.3%	3	2.8%	404	53.5%
Female	745	60.1%	289	76.7%	105	97.2%	351	46.5%
Rapid test								
IgG positive	257	20.7%	56	14.9%	17	15.7%	184	24.4%
IgG negative	983	79.3%	321	85.1%	91	84.3%	571	75.6%
Symptoms among IgG +								
At least one	192	74.7%	44	78.6%	12	70.6%	136	73.9%
No symptoms	65	25.3%	12	21.4%	5	29.4%	48	26.1%

All the participants were tested to detect IgG and IgM antibodies against SARS-CoV-2 in April 2020. However, this brief report addresses only the data on IgG antibodies. The global prevalence of IgG antibodies was 20.7% (257). Paediatricians and their staff members reported a similar prevalence of 14.9% (56) and 15.7% (17), respectively. A higher prevalence of 24.4% (184) was reported among the cohabitant relatives (Table 1). It is interesting to note that the prevalence of staff members with positive IgG was very similar to that of paediatricians. Moreover, cohabitant relatives of paediatricians reported a higher rate of IgG positive test than paediatricians themselves. These data suggest that doctors could have get infection not only from patients but also outside work.

Overall, 64.2% (796) of the participants reported close contacts with a suspected case of COVID-19, even though only 32.4% (258) of those cases were confirmed to be coronavirus infection. Specifically, 62.6% (236) of the paediatricians were exposed to a suspected case, later confirmed in 77.5% (183) of these cases. Their staff members and cohabitant relatives described similar exposures to suspected COVID-19 patients; however, the diagnosis was confirmed in only 35.4% (23) and 37.5% (182) of those cases, respectively. Occupational exposure was the main means of exposure for the paediatricians (72.9%) and their staff members (69.2%). Cohabitant relatives were exposed at the workplace in 44.0% of the cases, while 38.2% of these individuals reported having had close contact with a cohabitant relative affected by COVID-19.

As to the serological test results, a total of 185 out of 257 IgG positive study participants were exposed to close contact with a suspected case of Covid-19. The gap time in days between time of contact and test administration had an average of 45 days and a standard deviation of 13 days (q1 = 39 days, q3 = 52 days).

Overall, almost three in 10 participants had symptoms related to COVID-19 in the weeks before the tests. Interestingly, 79.8% (292) of the symptomatic participants had had previous close contact with a suspected COVID-19 case, while only 57.6% (504) of asymptomatic participants had had it.

More than 70% of study participants who tested positive related having demonstrated symptoms compatible with SARS-CoV-2 infection. Specifically, 78.6% of the paediatricians, 70.6% of the staff members, and 73.9% of the cohabitant relatives with a positive serological test reported symptoms (Table 1). The date of symptoms occurrence was available for 151 out of 192 IgG positive study participants who had symptoms. The gap time in days between time of contact with a suspected case of Covid-19 and symptoms onset had an average of 6.8 days and a standard deviation of 13.2 days (q1 = 6 days, q3 = 10 days).

The most common symptoms were fever (100%), anosmia (57.2%), ageusia (54.1%), gastrointestinal disorders (53.3%), shortness of breath (24.1%), and oxygen saturation < 95% (16.3%). Among the symptomatic participants, 4.9% (18) needed hospitalization due to the severity of their clinical conditions.

Polymerase chain reaction diagnostic tests on nasal swabs were performed in 26.6% (71) of the positive serology tests and 8.3% (82) of the negative serology tests. It was thus possible to estimate the performance characteristics of the used serological test. We found a relative sensibility of 96.0% and a relative specificity of 79.2%.

The Italian National Institute of Statistics (ISTAT) recently published a report on the seroprevalence of antibodies against SARS-CoV-2 in the Italian population [13]. According to ISTAT, about 2.5% of Italians have antibodies for the novel coronavirus, with a significant difference between regions. Indeed, about 50% of individuals with antibodies live in Lombardy, where the seroprevalence is around 7.5%, which is up to seven times higher than in other regions, where there was the lowest level of virus spreading. Concerning HCWs, ISTAT reported that their seroprevalence is higher than that of the general population, reaching almost 10% in those regions that the pandemic has affected most.

Although these data were collected differently than those collected for this study, the ISTAT report is interesting and useful for interpreting the current results. This research indicates that about 15% of the paediatricians and staff members of paediatric clinics had IgG antibodies for SARS-CoV-2, which is higher than the percentages for the general population of Lombardy and other HCWs. This finding may suggest that paediatricians and their staff members have been highly exposed to the novel coronavirus, especially at the beginning of the pandemic, when knowledge of the virus was limited. Moreover, paediatricians’ relatives also had a higher seroprevalence than the general population. Hence, in this recent new peak of pandemic, it is important that paediatricians use correctly PPE and separate patients with covid-19 related symptoms from other patients, scheduling different appointments, as also suggested by the Italian Pediatric Society [14].

Finally, the current results confirm that the rapid tests used in this screening have a decent performance in terms of sensibility, while specificity stopped at 80%. However, the initiative of SIMPeF has been extremely useful in assessing the impact of the COVID-19 pandemic on paediatric settings and to raise awareness family paediatricians about the spreading of coronavirus, at a time when the world was faced with few certainties and many unknowns.

## Data Availability

The data were collected on the portal www.simpef.it and are in possession of the SIMPeF. The datasets used and/or analysed during the current study are available from the corresponding author on reasonable request.

## Abbreviations

COVID-19: Corona virus disease 2019
HCWs: Health care workers
IgG: Immunoglobulin G
IgM: Immunoglobulin M
ISTAT: Istituto nazionale di statistica (Italian national institute of statistics)
PCR: Polymerase chain reaction
PPE: Personal protective equipment
SARS-CoV-2: Severe acute respiratory syndrome – coronavirus – 2
SIMPeF: Sindacato medici pediatri di famiglia
